# A Pilot Study on the Efficacy of an App-Based Rehabilitation Counselling Program after Total Knee Arthroplasty

**DOI:** 10.3390/healthcare12131329

**Published:** 2024-07-03

**Authors:** Sang-Ho Han, Se-Hee Kong

**Affiliations:** 1 Department of Orthopedic Surgery, Daechan Hospital, 590 Inju-Daero, Namdong-Gu, Incheon Metropolitan City 21570, Republic of Korea; doctortrust@naver.com; 2 Hospital Daechan Sports Medical Research Center, 590 Inju-Daero, Namdong-Gu, Incheon Metropolitan City 21570, Republic of Korea

**Keywords:** app-based rehabilitation, rehabilitation, total knee arthroplasty, digital health care

## Abstract

The aim of this study was to determine the effects of a novel app-based rehabilitation counselling program in patients recovering from total knee arthroplasty. In the app-based rehabilitation counselling program, a counselor provides one-on-one rehabilitation exercises and management-focused psychological counselling after total knee arthroplasty (TKA). This study included 42 patients, who were divided into three groups of 14 patients each: Group A, whose rehabilitation training was based on a guideline leaflet; Group B, whose rehabilitation was based on the app-based rehabilitation counselling program; and Group C, whose rehabilitation training was based on continuous passive motion combined with the app-based rehabilitation counselling program. To determine the effects of rehabilitation, the isokinetic knee muscle strength and knee joint range of motion were measured in addition to knee function tests such as the Western Ontario and McMaster Universities Osteoarthritis Index (WOMAC) and the visual analogue scale (VAS) for pain at two and three months after rehabilitation. The comparison of the means of the three groups was analyzed using one-way analysis of variance (ANOVA), with Group C showing significant variance in isokinetic knee muscle strength (*p* < 0.05), passive ROM (*p* < 0.01), and WOMAC (*p* < 0.05) after three months. As a result, this study confirmed the positive potential of the app-based rehabilitation counselling program.

## 1. Introduction

Total knee arthroplasty (TKA) is generally performed in older adults aged ≥65 years [1,2]. It can generate successful outcomes, including relief from pain caused by knee osteoarthritis, improved physical functions, and increased quality of life [3,4,5].

However, postoperative rehabilitation training is required to recover muscle mass and strength, which are reduced as a result of limited joint activity before surgery, as well as to strengthen the newly structured joint [6,7,8]. Su et al. [9] stated that rehabilitation after TKA can have a significant impact on the surgical outcome.

Early rehabilitation immediately after TKA is critical for reducing knee pain, strengthening the quadriceps femoris for gait, reducing knee swelling, and restoring the normal range of motion (ROM) of the knee [8]. However, TKA cannot resolve the preoperative stiffness. Therefore, focus is placed on postoperative rehabilitation, and continuous passive motion (CPM) is included in the extensive rehabilitation program to restore normal ROM. The CPM device passively and repeatedly moves the knee at a set ROM, with known benefits including rapid functional recovery of the knee [10]. Another benefit of CPM is that it is elderly-friendly, as the participants can repeat the motions while checking their knee angles. The key to rehabilitation is continuous engagement. Therefore, new systems that enable patients to rehabilitate on their own at home after discharge are being developed. Accordingly, a new system is being developed that allows patients to rehabilitate themselves at home after discharge. This is based on the view that even after discharge, if patients have comprehensive and systematic information about their surgery and treatment, they can reduce anxiety by understanding their physical situation and participating in activities such as rehabilitation according to the information provided [11,12]. Additionally, the patient’s pain after TKR can entail a slow recovery, cause persistent pain and discomfort, and ultimately reduce the effectiveness of TKR [13,14,15]. Therefore, Pronk et al. [15] stated that pain should be controlled effectively both in the hospital and at home. With this trend of change, mobile app-based rehabilitation and remote online rehabilitation programs have been developed in recent years. For instance, Bäcker et al. [16] developed a knee trainer with app-based feedback control, whereby the app-to-sensor connection provided real-time feedback via Bluetooth as the patient performed exercises using a tablet PC, thereby allowing for step-by-step independent training. The program was designed for easy access to improve weakened quadriceps femoris and knee motions after surgery. The use of the app produces far more outstanding short-term functional outcomes, although additional education is necessary for the older adult population to ensure appropriate use.

For remote rehabilitation training, Correia et al. [17] developed a virtual remote conversation system that provides a conversation application to display the exercise performed for the patient, together with a biometric reader, which is managed by a central server. The system is characterized by a therapist who monitors the process via IVT and provides interventions during rehabilitation. However, mobile apps developed for rehabilitation mainly rely on video instructions alone, making it difficult to correct the exercise posture [18,19]. To compensate for this, Motion Coach (Kaia Health GmbH) developed a system that provides real-time, customized feedback on body positions during exercise using only a smartphone [20].

However, these mobile apps only provide rehabilitation management and measures but do not manage the patient’s emotional state during rehabilitation. Many studies have shown that psychological conditions such as anxiety, depression, and negative thinking affect surgical outcomes and recovery. As such, the patient’s emotions are very important in recovery, but an app that takes care of the patient’s emotions after surgery does not appear to have been developed yet according to a recent literature review.

Recently, Mecklenburg et al. [21] developed Hinge Health DCP, which provides muscle-strengthening activities, education, cognitive behavioral therapy, and psychosocial support for patients with chronic pain in the musculoskeletal system, and the use of this program was said to be effective. Although the subject was not a patient with TKA, the program included cognitive behavioral therapy. However, it lacked one-on-one counselling.

**Figure 1:**
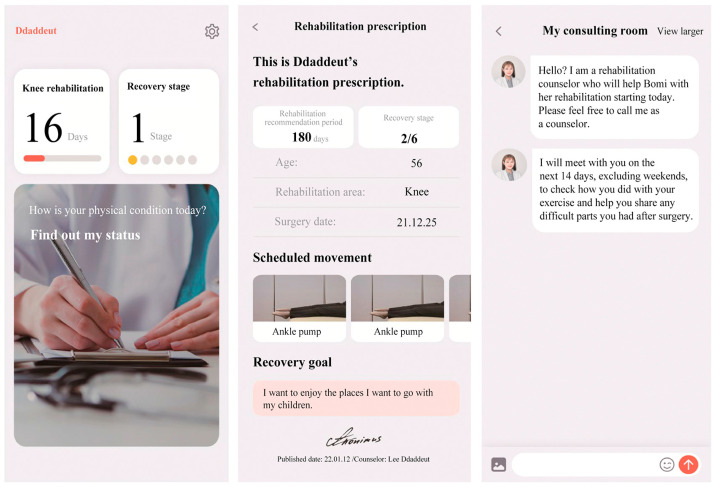
App-based rehabilitation counselling programs.

**Figure 2:**
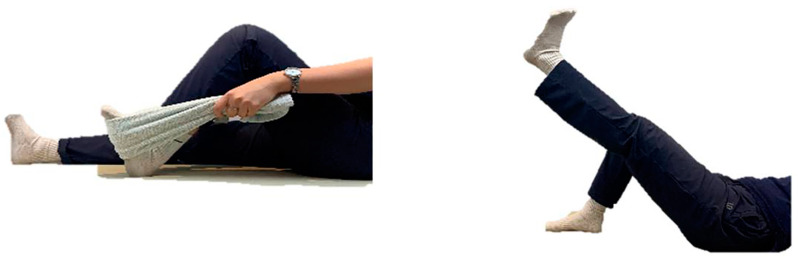
Example of rehabilitation exercises.

Recently, a new app-based rehabilitation counselling program called ‘Ddaddeut’ was introduced, as presented in Figure 1. This program was developed by One Medics, Inc., Seoul, Republic of Korea. The program is an individualized rehabilitation assistance program that helps patients check their physical state every day and undergo structured rehabilitation exercises suggested by the program based on their state (Figure 2). Patients had access to professional counsellors through the app, and they also received emotional counselling addressing their postoperative anxiety and depression so that they could adhere to and complete rehabilitation. In particular, TKR patients are older and may face many difficulties in rehabilitation, so we expect that management through real-time communication and emotional care will be of great help. The key goals of this study were to apply this novel app-based rehabilitation counselling program to post-TKA patients and to explore its effects. As such, the researchers hypothesized that patients who utilize both CPM and the app-based rehabilitation counselling program would experience better effects than other groups.

## 2. Materials and Methods

This quasi-experimental study included patients who underwent TKA at D hospital in Incheon, South Korea, and participated in post-TKA rehabilitation between December 2020 and April 2022. The sample size was estimated using G-Power 3.1 in the conditions for one-way analysis of variance (ANOVA) set as follows: a 0.05 significance level, 0.8 testing power, and moderate effect size. The total sample size was 42.

Using Microsoft Excel 2010, patients were randomized into three groups. In particular, the method involved formulating random numbers using the RAND function to create the groups. Then, rankings were set, and the groups were assigned by rank.

The groups were defined as follows: Group A (rehabilitation training based on a guideline leaflet), Group B (rehabilitation based on the app-based rehabilitation counselling program), and Group C (rehabilitation training based on CPM combined with the app-based rehabilitation counselling program).

The criteria for the TKA surgical site were based on primary unilateral TKA. Patients who had undergone reoperation were excluded. The demographic details of the patients and their BMIs are presented in Table 1. Patients were asked to follow the progression of exercise according to the rehabilitation training group to which they belonged and comply with the respective instructions until 3 months after surgery. The experimental procedures involved post-TKA patients in all three groups. The isokinetic knee muscle strength and knee ROM were measured, and a knee function test known as the Western Ontario and McMaster Universities Osteoarthritis Index (WOMAC) and the visual analogue scale (VAS) for pain were used for evaluations at 2 and 3 months after surgery. This study was approved by the Institutional Review Board of Sangmyung University (protocol code: SMUIRB Ex-2022-010; date of approval: 7 April 2022).

### 2.1. Rehabilitation Group Sessions

The rehabilitation training program for each group is shown in Table 2. The programs were based on the rehabilitation program suggested by Aseer et al. [22], with modifications made by an orthopedic surgeon, physical therapist, and exercise therapist. The programs consisted of Phases 1–3 during weeks 1–12 after surgery.

The app-based rehabilitation counselling program is a total care program providing rehabilitation training and management, as well as emotional care by a professional counsellor.

Patient management is handled directly by the counsellor, while the patients are trained for rehabilitation by a therapist. Based on the rehabilitation exercises shown in the table above, the stages can go from 1 (1 month) to 6 (6 months). In this study, the exercises were conducted for up to three months.

Prior to participating in the app-based rehabilitation program, patients were guided to self-diagnose and enter data regarding their level of pain, swelling, heat sensation, and traction. Then, through an analysis of the patient’s current status, the app offered suitable rehabilitation training, which consisted of four to six exercises involving stretching, muscle strengthening, and joint ROM. Each exercise was displayed in step-by-step images to allow patients to follow the instructions with ease. After the exercises, the patients were guided to self-check their progression and enter the data. Based on this system, the level of patient rehabilitation could be primarily managed by the counsellor. Subsequently, through one-on-one conversations, the challenges that patients faced during rehabilitation training were identified, or the exercise intensity was adjusted. In addition, this program taught relaxation and breathing methods to relieve depression and provided psychological rehabilitation counselling when necessary.

CPM involves the use of an isokinetic device for exercise, which is mostly used immediately after surgery and is the standard therapy provided at numerous institutions. It aims to prevent joint ROM limitations and improve ROM and knee joint functions. Patients in Group 3 had the device installed at home while they were educated on using the device and exercise methods. The CPM requires the control of angles as follows: 0°–100° for 2–3 times per week for weeks 1–2 and 0°–140° up to weeks 3–6. The patients were guided to consult a professional counsellor via an app-based rehabilitation counselling program regarding any questions they might have during the training.

### 2.2. Measurements

The instruments used to determine the effects of rehabilitation across the three groups with respect to pre-test–post-test variation in the follow-up test at two and three months after surgery were as follows: First, the isokinetic knee muscle strength was measured using an isokinetic dynamometer (Biodex System 4 model; Biodex Medical Systems, Inc., New York, NY, USA). Muscle strength was measured for the peak torque of the knee joint at flexion and extension at angular speeds of 60°/s and 180°/s. Passive ROM was measured by the therapist and defined as the maximum value of the knee joint at 90° flexion using a standard portable protractor. The perceived, subjective functional status of patients was assessed using the WOMAC [23] with a score range of 0–100, with higher scores indicating poorer outcomes. The level of pain was measured using 10 cm VAS; a score of 0 on the left end of a horizontal line indicated ‘no pain’, and a score of 10 on the right end of the line indicated ‘unbearable pain’ [24]. The patient was guided to score their knee pain.

### 2.3. Statistical Analysis

For data analysis, the SPSS statistical package (SPSS, version 22.0, Chicago, IL, USA) was used. Mean (M) and standard deviation (SD) of study subjects were estimated for each group, and a one-way ANOVA was conducted to confirm whether the average of the rehabilitation results would be different for any of the three groups. Post hoc analysis was then performed to compare the differences in the results of the groups using a test called Scheffe’s method. Statistical significance was set at *p* < 0.05. Missing data were excluded from the analysis.

## 3. Results

The result of isokinetic knee muscle strength at 60°/s and 180°/s are presented in Table 3. The 60°/s peak torque at extension and flexion did not vary significantly across the three groups before surgery. However, the variation in Groups B and C’s extension and flexion was significant after two and three months (*p* < 0.05). A Scheffe’s post hoc analysis was performed to examine the detailed results. The results showed that Group C showed significant variation two and three months after surgery.

For the 180°/s peak torque at extension, all groups displayed no significant variation before and two and three months after surgery. However, there was a significant variation at flexion three months after surgery in Group C (*p* < 0.05). In order to verify in which group there was a difference, a Scheffe’s post-test was also conducted, and the result showed that there was a difference between A and C. As with the previous results, it can be seen that Group C has more variation than Group A.

**Figure 3:**
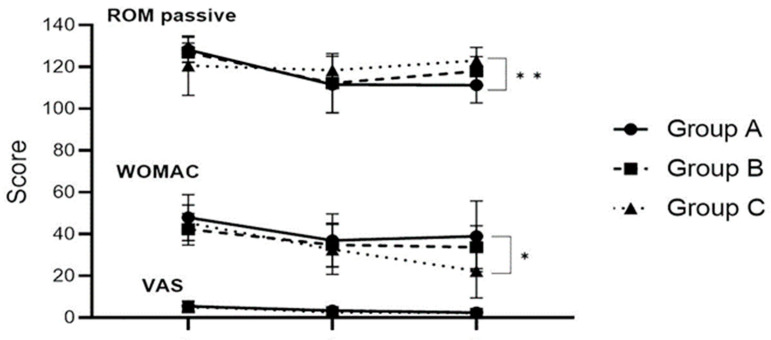
Comparison of the clinical and physical function scores of Groups A, B, and C during preoperation, two months postoperation, and three months postoperation, *p* < 0.05 *, *p* < 0.01 **.

The results of the clinical and physical function scores are presented in Table 4. The variation in passive ROM showed a significant variance in Group C three months after surgery (*p* < 0.01). As a result of Scheffe’s post hoc analysis, there was significant change in Groups A and C, and the results of Group C can be interpreted as being better. For WOMAC, the results showed no significant variation before and two months after surgery, although a significant variation was found three months after surgery in group three (*p* < 0.05). Scheffe’s post hoc tests revealed that Group C had a significantly improved WOMAC score compared with Group A. With regard to the VAS score for pain and VAS evaluation of pain, the results showed no significant variation before and two and three months after surgery across all groups (Figure 3).

## 4. Discussion

TKA is a common, standardized surgery for patients with knee discomfort, for which postoperative rehabilitation is critical to ensure rapid and stable recovery. The methods of rehabilitation have steadily improved, and the advancement of technology has allowed for the recent development of app-based rehabilitation programs to assist with accessible professional rehabilitation, even at home.

This program provides guidance on exercise methods and management, as well as emotional care, for the patient’s continuous rehabilitation after discharge.

First, the isokinetic knee muscle strength was measured to verify the effects of the rehabilitation training program. Two and three months after surgery, the peak torque at 60°/s and 180°/s was high, with the most outstanding result exhibited by the group that used CPM in combination with the app-based rehabilitation counselling program.

This is a key part of rehabilitation, because isokinetic knee muscle strength helps stabilize the knee. Moreover, as it can only be improved through exercise, the key is to exercise consistently. Because patients with TKA tend to be older, they are inevitably less able to explore and consistently exercise independently. This also applies to CPM using machines. Therefore, it is believed that the app-based rehabilitation counselling program’s systematic guidance on exercise methods and management ensured accurate CPM and continuous knee exercise for the patients. The knee’s ROM was also measured to verify recovery, and a significant change was observed three months after surgery in Group C.

The ROM also requires consistent exercise to soften stiff knees after surgery. In particular, this is an essential part of returning to daily life post-surgery, because the ROM angle must be restored to some extent before some actions, such as going up and down the stairs, become comfortable. As the ROM is important, more systematic rehabilitation exercise provision and management were needed, and the patient’s active practice would have helped recover ROM. There will also be effects brought about by CPM, such as exercising at different knee angles. However, this result was inconsistent with a study by Bäcker et al., which showed a lack of significant variation between active and passive ROM measured after the post-TKA app-based rehabilitation training program [16]. The ROM in this study did not show statistical significance in the difference between the app and the control groups, but the average score showed that the app-based rehabilitation training program had a high score, suggesting that the app group may have had some positive influence, which aligns with the positive results of the study. In addition, many studies measured active ROM rather than passive ROM, making accurate comparison difficult.

WOMAC, which was used to assess the overall knee function, showed no significant variation before and two months after surgery, whereas a significant variation was found three months after surgery. To assess which group displayed variation after three months of rehabilitation, a post hoc test was performed. The results showed that Group C had significant variation compared to Group A. In general, positive effects of rehabilitation were observed three months after surgery, but it is believed that the active rehabilitation would have enabled the patient to quickly return to their daily life. The active assistance provided by the app-based rehabilitation counselling program appears to have had greater effects than the self-guided program. Furthermore, a synergistic effect was detected using a device to assist early rehabilitation. In a previous study by Piqueras et al. [25], no significant variation in the WOMAC score was found after three months between the standard rehabilitation and interactive virtual telerehabilitation groups, which is inconsistent with the results of this study. However, because the WOMAC score measures the general functional status of the knee, it appears that this can also be improved with a certain amount of exercise. Therefore, while it is inconsistent with the study results, it should be noted that the functional status of the knee was enhanced through rehabilitation.

VAS, which was used to assess pain at two and three months after rehabilitation, showed no significant variations. This is consistent with a study by Correia et al. [17], which found no significant changes 3 months after surgery between the conventional and remote rehabilitation groups. Duong et al. [26] examined the effect of rehabilitation after TKR in a digital technology package group consisting of an exercise app fitness tracker and management by an online health manager and a group with a fitness tracker but no follow-up management. A numerical rating scale (NRS) was used to check the pain results. Pain was clinically reduced by three times in the group that used the digital package after rehabilitation, but this was not statistically significant. This result is also consistent with the results of this study.

We examined the effect of rehabilitation after TKR in a digital technology package group consisting of an exercise app fitness tracker and management by an online health manager and a group with a fitness tracker but no follow-up management. Although there were no statistically significant changes in the three groups, there was a decrease in pain in the scores, so all rehabilitation exercises can be considered meaningful. As a result, it is believed that it is important to help patients continue to exercise, because all rehabilitation exercises after TKR helped improve pain.

To summarize, in comparison with various previous studies, knee function or pain may improve depending on the patient’s efforts, even if general rehabilitation is performed. However, the recovery of knee strength or ROM requires steady rehabilitation, so continuous care is required. The app-based rehabilitation counselling program appears to be helpful. In particular, better results were obtained when CPM was used in combination rather than when the app-based rehabilitation counselling program was solely conducted. Furthermore, although it was not statistically significant, the score of Group B in the app-based rehabilitation counselling program was better than that of Group A, who underwent rehabilitation training based on a guideline leaflet.

For successful rehabilitation after surgery, rehabilitation methods and tools that are well suited to the patient are necessary. In addition, for continuous rehabilitation, it is important to have a stable psychological state, willingness to rehabilitate, and sincerity in carrying out rehabilitation. The app-based rehabilitation counselling program supplemented this to some extent through one-on-one consultations with patients and experts.

However, in this study, the effects of care from a counsellor on patients’ anxiety and depression could not be measured. Nevertheless, several patients were interviewed to check the related level of satisfaction. The results can be summarized as follows: patients worried about postoperative side effects before surgery and thought that surgery would be the end of treatment. However, as they became aware of the importance of rehabilitation in the process of recovery, they vaguely considered postoperative rehabilitation. The severe pain after surgery had particularly made them regret undergoing the surgery. However, one-on-one conversations with the counsellor, who educated them on the process of recovery and plausible symptoms, helped them achieve emotional stability. A patient who underwent one-on-one counselling said in an interview that ‘I’ve arrived home from the hospital. The message they sent gave me strength and put my mind at ease. It touched me a lot. I didn’t know much about exercise, but it was helpful because they told me what exercises I should do and sent me pictures. I was especially anxious because the pain was so severe, but I felt reassured when they explained that it was part of the process of getting better’. The app-based rehabilitation counselling program went beyond the simple provision and management of rehabilitation and ensured that the patients could receive care for the different emotional states they underwent, from surgery to rehabilitation. In addition, modern society is continuously ageing with increased mobility, and there is a need for easily accessible rehabilitation programs without temporal or spatial restrictions and a platform that can address any questions regarding the process of rehabilitation. Therefore, an app-based rehabilitation counselling program would be useful for post-TKA patients.

This study has several limitations. First, the number of patients in each group was 14, which was defined as a pilot study. There were difficulties in recruiting study subjects in the three groups. However, the research results can be treated as a basic experiment. Second, the fact that the number of participants in the study was limited to patients who underwent surgery at one hospital may lead to potential bias in the data, but because the study analyzes rehabilitation results after discharge, it is unlikely to be a major problem. In addition, the self-reporting method was selected for some of the score measurements, and because the researcher in charge explained this concept ahead, the respondents were able to respond objectively.

Third, the effects of direct counselling on the emotional state of a patient could not be measured. As such, it is crucial to determine the emotional state of the patient before and after rehabilitation, as well as their satisfaction in using the app.

Finally, rehabilitation proceeded at a slower pace when the patient was relatively unresponsive to the app-based rehabilitation counselling program.

In the future, even if the number of rehabilitation groups is adjusted, after securing more people, follow-up research must be conducted by supplementing and incorporating the functional status of rehabilitation, the psychological state of patients during rehabilitation, their satisfaction with the rehabilitation app, and their app usage frequency.

Despite these limitations, this study serves as basic research that lays the foundation for the widespread use of an app-based rehabilitation counselling program, which was used for the first time in this study after TKA.

## 5. Conclusions

In conclusion, the efficacy of app-based rehabilitation programs, professional counselling, and their combination was tested for the first time in this study, and their potential for use was confirmed through short-term follow-up monitoring. The combination of the app-based rehabilitation counselling program ‘Ddaddeut’ with a professional rehabilitation device, CPM, could further improve its efficacy. Ultimately, it appeared to be as effective as other rehabilitation methods. The counselling effect of the program was also verified through interviews with the patients.

## Figures and Tables

**Table 1:** Patients’ demographic data.

Variable	Group A (n = 14)	Group B (n = 14)	Group C (n = 14)	*p* Value
Age (yr)	69.54 ± 6.63	69.08 ± 6.09	66.38 ± 7.26	n.s.
Height (cm)	155.77 ± 8.69	159.00 ± 6.81	164.08 ± 7.51	n.s.
Weight (kg)	63.46 ± 9.79	66.54 ± 11.82	71.31 ± 12.33	n.s.
BMI: Weight (kg)/Height (m^2^)	26.09 ± 2.82	26.18 ± 3.26	26.44 ± 4.11	n.s.

Values are presented as mean (SD); *p* < 0.05; n.s.: not significant; BMI: body mass index.

**Table 2:** Rehabilitation group protocols.

	Index	Exercise	Intensity
Rehabilitation training based on a guideline leaflet (A)	1–2 weeks	Ham stretching	10 reps × 3 sets
	60 min, 3 times a week	Ankle pump	100 reps
		Knee extension stretching	0°–90°/50 reps
		Front SLR	10 reps × 3 sets
		Side SLR	10 reps × 3 sets
		Back SLR	10 reps × 3 sets
		SAQ	10 reps × 3 sets
		2 LAQ	10 reps × 3 sets
		Calf raise	10 reps × 3 sets
		Chair squat	10 reps × 3 sets
	3–6 weeks	Band ham stretching	(10 s × 10 reps) × 3 sets
	60 min, 3 times a week	Ankle pump	0°–90°/50 reps
		Knee extension stretching	10 reps × 3 sets
		Pocket front SLR	10 reps × 3 sets
		Pocket side SLR	10 reps × 3 sets
		Pocket back SLR	10 reps × 3 sets
		Pocket SAQ	10 reps × 3 sets
		Pocket LAQ	10 reps × 3 sets
		Calf raise	10 reps × 3 sets
		Viking walking exercise	10 reps × 3 sets
		Wall squat	10 reps × 3 sets
	7–12 weeks	Band ham stretching	10 reps × 3 sets
	60 min, 3 times a week	Foam roller quad release	50 reps
		Foam roller TFL release	50 reps
		Foam roller and release	50 reps
		Weight knee extension	10 reps × 3 sets
		Single stand hip flex	10 reps × 3 sets
		One leg squat	10 reps × 3 sets
		One leg Y squat	10 reps × 3 sets
		Cool down	
App-based rehabilitation counselling program (B)	Until 12 weeks after discharge	Same as the above exercises Through 1:1 messenger, a professional counsellor checks the exercise method and progress according to the individual and encourages carrying out the exercises	
CPM, App-based rehabilitation counselling program (C)	1–2 weeks 20–30 min, 3 times a week	0°–100°	
	3–6 weeks 20–30 min, 3 times a week Until 12 weeks Same as the above exercises	0°–140°	

**Table 3:** Results for isokinetic knee muscle strength.

	Preoperation				Postoperation, 2 Months				Postoperation, 3 Months				
	Group A (n = 14)	Group B (n = 14)	Group C (n = 14)	*p* Value ^†^	Group A (n = 14)	Group B (n = 14)	Group C (n = 14)	*p* Value ^†^	Group A (n = 14)	Group B (n = 14)	Group C (n = 14)	*p* Value ^†^	*p* Values ^‡^ (Post Hoc by Scheffe)
Muscle torque at 60°/s													
Peak torque at extension (N-m)	34.55 ± 20.17	60.46 ± 37.09	54.79 ± 6.91	0.845	31.48 ± 12.53	46.78 ± 35.96	66.21 ± 400.01	0.041 *	48.20 ± 20.51	60.78 ± 27.09	78.34 ± 40.48	0.043 *	A < C
Peak torque at flexion (N-m)	34.95 ± 13.82	26.93 ± 21.44	30.49 ± 27.06	0.667	14.76 ± 12.54	32.66 ± 26.37	42.24 ± 29.08	0.014 *	38.07 ± 26.27	28.61 ± 16.91	63.23 ± 44.95	0.018 *	A < C
Muscle torque at 180°/s													
Peak torque at extension (N-m)	47.65 ± 14.71	42.81 ± 25.60	37.45 ± 34.57	0.644	20.27 ± 15.65	36.87 ± 31.29	39.63 ± 27.15	0.109	44.79 ± 19.93	36.44 ± 15.23	62.01 ± 46.49	0.090	
Peak torque at flexion (N-m)	24.44 ± 12.60	18.54 ± 14.33	21.50 ± 22.25	0.698	10.54 ± 10.88	20.81 ± 23.40	22.88 ± 22.25	0.219	28.21 ± 20.07	18.35 ± 12.56	49.29 ± 36.41	0.008 **	A > C

^†^ *p* values were calculated with the use of one-way analysis of variance. ^‡^ *p* values were calculated with the use of post hoc analysis by the Scheffe method. *p* < 0.05 *, *p* < 0.01 **.

**Table 4:** Results for clinical and physical function scores.

	Preoperation				Postoperation, 2 Months				Postoperation, 3 Months				
	Group A (n = 14)	Group B (n = 14)	Group C (n = 14)	*p* Value ^†^	Group A (n = 14)	Group B (n = 14)	Group C (n = 14)	*p* Value ^†^	Group A (n = 14)	Group B (n = 14)	Group C (n = 14)	*p* Value ^†^	*p* Values ^‡^ (Post Hoc by Scheffe)
ROM passive	128.13 ± 5.94	126.82 ± 4.62	120.56 ± 14.24	0.195	111.50 ± 13.55	112.14 ± 14.10	118.33 ± 2.42	0.449	111.25 ± 8.56	117.86 ± 6.99	123.00 ± 6.33	0.004 **	A < C
WOMAC	47.91 ± 10.99	42.29 ± 7.63	45.31 ± 8.48	0.307	36.91 ± 12.68	34.77 ± 10.36	32.62 ± 11.94	0.669	38.91. ± 16.89	33.69 ± 10.25	22.38 ± 12.95	0.012 *	A > C
VAS	5.45 ± 2.42	5.00 ± 1.18	5.31 ± 1.93	0.822	3.36 ± 1.80	3.46 ± 1.27	2.54 ± 1.39	0.240	2.38 ± 1.94	2.15 ± 1.14	2.23 ± 1.83	0.938	-

Values are presented as mean (SD), *p* < 0.05 *, *p* < 0.01 **; ROM: range of motion; WOMAC: Western Ontario and McMaster Universities Osteoarthritis Index; VAS: visual analogue score. ^†^ *p* values were calculated with the use of one-way analysis of variance. ^‡^ *p* values were calculated with the use of post hoc analysis by the Scheffe method.

## Data Availability

The datasets analyzed during the current study are publicly available (https://doi.org/10.6084/m9.figshare.25592088).
